# The Need for Randomization in Animal Trials: An Overview of Systematic Reviews

**DOI:** 10.1371/journal.pone.0098856

**Published:** 2014-06-06

**Authors:** Jennifer A. Hirst, Jeremy Howick, Jeffrey K. Aronson, Nia Roberts, Rafael Perera, Constantinos Koshiaris, Carl Heneghan

**Affiliations:** 1 Nuffield Department of Primary Care Health Sciences, University of Oxford, Oxford, United Kingdom; 2 Bodleian Libraries, University of Oxford, Oxford, United Kingdom; McGill University, Canada

## Abstract

**Background and Objectives:**

Randomization, allocation concealment, and blind outcome assessment have been shown to reduce bias in human studies. Authors from the Collaborative Approach to Meta Analysis and Review of Animal Data from Experimental Studies (CAMARADES) collaboration recently found that these features protect against bias in animal stroke studies. We extended the scope the work from CAMARADES to include investigations of treatments for any condition.

**Methods:**

We conducted an overview of systematic reviews. We searched Medline and Embase for systematic reviews of animal studies testing any intervention (against any control) and we included any disease area and outcome. We included reviews comparing randomized versus not randomized (but otherwise controlled), concealed versus unconcealed treatment allocation, or blinded versus unblinded outcome assessment.

**Results:**

Thirty-one systematic reviews met our inclusion criteria: 20 investigated treatments for experimental stroke, 4 reviews investigated treatments for spinal cord diseases, while 1 review each investigated treatments for bone cancer, intracerebral hemorrhage, glioma, multiple sclerosis, Parkinson's disease, and treatments used in emergency medicine. In our sample 29% of studies reported randomization, 15% of studies reported allocation concealment, and 35% of studies reported blinded outcome assessment. We pooled the results in a meta-analysis, and in our primary analysis found that failure to randomize significantly increased effect sizes, whereas allocation concealment and blinding did not. In our secondary analyses we found that randomization, allocation concealment, and blinding reduced effect sizes, especially where outcomes were subjective.

**Conclusions:**

Our study demonstrates the need for randomization, allocation concealment, and blind outcome assessment in animal research across a wide range of outcomes and disease areas. Since human studies are often justified based on results from animal studies, our results suggest that unduly biased animal studies should not be allowed to constitute part of the rationale for human trials.

## Introduction

### Bias in Animal Studies

Clinical epidemiologists and proponents of evidence-based medicine (EBM) have been using methods to reduce bias in human studies for over four decades. [1]–[5] Random allocation of participants to treatment groups, concealing the allocation sequence from those assigning participants to intervention groups (allocation concealment), and blinding of investigators assessing outcomes are now viewed as fundamental ways of ensuring quality and minimizing bias in clinical trials. [6] This is because concealed random allocation reduces selection bias and blinding outcome assessors reduces detection bias. [5] Armed with these methods, researchers have exposed several common medical practices as ineffective. For example, observational studies led us to believe that sodium fluoride reduced vertebral fractures, [7] that vitamin E reduced major coronary events, [8] and that high-dose aspirin was more effective than low-dose aspirin. [9] But subsequent randomized trials exposed all these treatments as useless or harmful. [10], [11] Benefits of randomization, allocation concealment, and blinding have been confirmed in larger meta-epidemiological studies. In the earliest of these, Schulz et al. (1995) found that odds ratios were exaggerated by 30% in trials lacking allocation concealment and by 17% in studies that lacked blind outcome assessment. [12] Subsequent larger investigations have confirmed these results and also shown that adequate randomization reduces bias in human studies. [13], [14]

A growing body of evidence is beginning to suggest that randomization, allocation concealment, and blinding outcome assessment can also reduce the risk of bias of animal studies. [15]–[25] Some researchers hypothesize that avoidable biases in animal studies contribute to the failure to translate much experimental work for human benefit. [26], [27] For example, while 503 of 835 candidate drugs for use in the management of stroke appeared effective in animal models, only one (tissue plasminogen activator) has proved sufficiently efficacious in humans. [28]

Much research into the empirical dimensions of bias in animal studies has been conducted by investigators from the Collaborative Approach to Meta Analysis and Review of Animal Data from Experimental Studies (CAMARADES) group. [29] CAMARADES researchers recently conducted an overview of systematic reviews of animal studies researching treatments for experimental stroke, and showed that failure to conceal allocation (but not failure to randomize or blind) exaggerated apparent treatment benefits in animal studies. [30] Despite this research, evidence-based principles have not yet been widely adopted in animal research; a recent study showed that only one in six controlled animal studies use randomization and only one in five use blind outcome assessment [31]. We therefore aimed to replicate the CAMARADES study independently and to expand its scope to include all conditions.

## Methods

We conducted an overview of systematic reviews. The protocol (unpublished) was finalized by JH, CH, RP, and JA in October 2012. We modified the protocol once to add the secondary analysis (testing the “unpredictability paradox”; see below). We searched MEDLINE and Embase databases (19 April 2012) and scanned reference lists for systematic reviews of animal studies that measured effects of randomization, allocation concealment, or blinding of outcome assessment. We included reviews in any disease area, using any intervention, any control group, any outcome measure and any animal model. We limited our search to the last 20 years and excluded human studies (search strategy in Appendix S1). We also excluded conference papers, studies not reported in English, ecological studies, and epidemiological studies.

Two reviewers (JH and JAH) independently extracted data on numbers of studies, numbers of animals, disease/condition, outcomes, effect measures, and effect sizes with confidence intervals, using piloted data extraction forms. Disagreements were resolved by discussion with other authors. Authors were contacted to request data which were not reported. To enable inclusion of one review [32] we estimated the number of animals in randomized and non-randomized groups by calculating the mean number of animals per study. To test whether this estimation affected our results we carried out a sensitivity analysis by removing the study from the meta-analysis. We assessed the risk of bias of included systematic reviews using the Assessment of Multiple Systematic Reviews (AMSTAR) criteria. [33]

We pooled results using the DerSimonian and Laird random effects model. [34] We reported outcomes for which differences between randomization/no randomization, allocation concealment/no allocation concealment, and blinding/no blinding were reported. We combined different outcomes and measurement units using standardized mean differences (SMDs), and quantified heterogeneity using the I-squared statistic. [35] We used meta-regression in a post-hoc analysis to examine whether various features influenced outcomes. Specifically, we investigated whether study size, disease state (stroke versus all other outcomes), or outcome measure were significantly associated with the effect size or could explain some of the heterogeneity.

For our secondary analysis we investigated the “unpredictability paradox”, which was proposed in a similar study involving human subjects. [13] The paradox states that the difference between inadequately randomized and randomized studies, although real, is unpredictable in terms of direction. This is plausible, given that the direction of bias may relate to differences in expected results. To investigate the paradox we ignored direction to see whether there was an absolute difference between results in randomized and non-randomized studies. We used the same method to investigate the unpredictability paradox for adequate allocation concealment and blinding. This approach is useful only as a guide, since with a large enough sample some absolute difference is likely to arise by chance alone.

## Results

**Figure 1:**
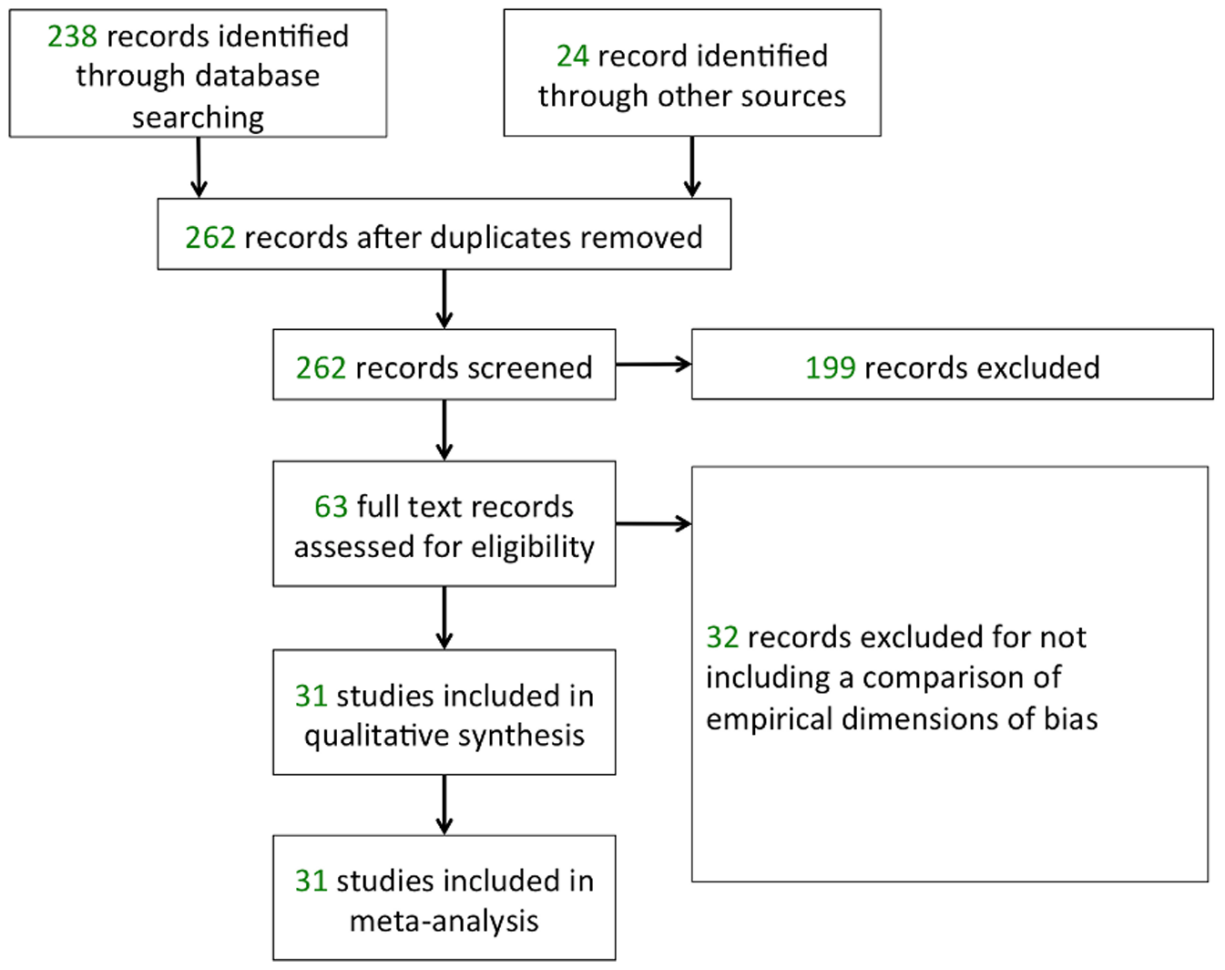
Flowchart of identified and included studies.

We identified 238 articles from our electronic search, and a further 24 articles by hand searching references and contacting CAMARADES authors. Two authors (JH, JAH) excluded 199 articles after reading titles and abstracts. We assessed the full text of the remaining 63 articles and excluded a further 32 for not including outcome data. CAMARADES authors generously shared data from 19 reviews in which data were not included in the published reports. We were left with 31 systematic reviews involving 7339 comparisons (estimated 123,437 animals) to include in the meta-analysis (see Figure 1). Characteristics of the 31 included reviews are shown in Table 1, and our data are available freely from the authors.

**Table 1:** Outcome measures, interventions, diseases, and effect sizes in included studies.

							Effect size (95% CI)		
Author	Intervention	Disease	Outcome measure	Number of comparisons	Number of animals	Randomized	Allocation concealed	Blinded	Animals used
**Antonic (2013)**	Stem cells Transplantation	Spinal Cord Diseases	Neurobehaviour score	315	5781	0.07 (0.01, 0.12)	0.08 (−0.01, 0.16)	−0.07 (−0.12, −0.02)	Rats, mice
**Banwell (2009)**	IL1 RA	Stroke	Infarct volume	44	784	−0.14 (−0.30, 0.02)	−0.22 (−0.38, −0.06)	0.05 (−0.10, 0.20)	Rats, mice
**Batchelor (2013a)**	Decompression	Spinal Cord Diseases	Neurobehaviour score	79	874	−0.09 (−0.23, 0.05)	0.15 (−0.06, 0.35)	−0.19 (−0.32, 0.05)	Dogs, mice, rats, sheep
**Batchelor (2013b)**	Hypothermia	Spinal Cord Diseases	Neurobehaviour score	25	448	−0.02 (−0.21, 0.17)	0.00 (−0.21, 0.22)	−0.04 (−0.23, 0.14)	Dogs, monkeys, rats
**Bath (2009)**	NXY-059	Stroke	Infarct volume	13	275	−0.25 (−0.49, −0.01)	−0.11 (−0.29, 0.08)	−0.14 (−0.33, 0.05)	Rats, mice, marmosets
**Bebarta (2003)**	Emergency medicine (all)	Any	Any outcome	290	*	−0.68 (−1.01, −0.29)	−	−0.66 (−1.13, −0.29)	Any (not specified)
**Currie (2013)**	Any	Bone cancer	Behavioural	202	4272	0.02 (−0.06, 0.10)	−	0.06 (−0.00, 0.12)	Rats, mice
			Histology, biochemistry	197	3228	−0.84 (−1.00, −0.68)	−	0.21 (0.13, 0.29)	
			Anatomical	27	470	−	−	0.08 (−0.10, 0.26)	
**Egan (2014)**	Exercise	Stroke	Neurobehaviour score	42	771	0.04 (−0.11, 0.18)	−0.02 (−0.17, 0.13)	−0.12 (−0.27, 0.02)	Rats, mice
			Infarct volume	65	987	0.13 (−0.01, 0.28)	−0.03 (−0.16, 0.10)	0.21 (0.02, 0.39)	
**Frantzias (2011)**	All drugs	Intracerebral hemorrhage	Neurobehaviour score	223	3932	−0.11 (−0.18, −0.05)	−0.09 (−0.18, −0.01)	0.02 (−0.05, 0.08)	Rats, mice, cats, rabbits, non−human primates
**Gibson (2006)**	Estrogen	Stroke	Infarct volume	22	372	0.49 (0.19, 0.79)	−	−	Rats, mice
**Hirst (2013)**	Temozolomide	Glioma	Median survival	123	2242	−0.49 (−0.57, −0.40)	−	−	Rats, mice
			Tumour volume	26	409	−0.01 (−0.25, 0.22)	−	0.16 (−0.17, 0.49)	
**Horn (2001)**	Nimodipine	Stroke	Infarct volume	7	121	−	−	0.16 (−0.23, 0.55)	Rats, rabbits, cats
**Janssen (2010)**	Enriched environment	Stroke	Learning	8	130	0.47 (0.11, 0.83)	−	−	Rats, mice
**Jerndal (2010)**	Erythropoietin	Stroke	Neurobehaviour score	29	489	−0.17 (−0.35, 0.01)	−0.21 (−0.46, 0.03)	−0.33 (−0.51, −0.15)	Rats, mice, gerbils
			Infarct volume	23	336	−0.27 (−0.48, −0.05)	−0.07 (−0.31, 0.16)	−0.17 (−0.38, 0.04)	
**Lees (2012)**	Stem cells	Stroke	Neurobehaviour score	233	3288	0.00 (−0.07, 0.07)	−0.01 (−0.09 to 0.07)	−0.03 (−0.10, 0.04)	Not specified
			Infarct volume	227	2804	−0.13 (−0.20, −0.05)	0.02 (−0.08, 0.11)	0.03 (−0.05, 0.11)	
**Macleod (2004)**	Nicotinamide	Stroke	Neurobehaviour score	52	711	−0.05 (−0.29, 0.19)	−	−0.05 (−0.29, 0.19)	Rats, mice
			Infarct volume	57	719	0.08 (−0.10, 0.27)	−	−0.01 (−0.17, 0.15)	
**Macleod (2005a)**	Melatonin	Stroke	Neurobehaviour score	6	47	−	−	−0.10 (−0.47, 0.28)	Rats, mice
			Infarct volume	27	419	0.20 (−0.02, 0.42)	0.31 (0.04, 0.58)	0.00 (−0.26, 0.27)	
**Macleod (2005b)**	FK 506 (tacrolimus)	Stroke	Neurobehaviour score	8	82	−0.89 (−1.35, −0.43)	−	−	Rats, mice, monkeys, gerbils
			Infarct volume	95	1569	0.10 (−0.06, 0.26)	−	0.12 (−0.01, 0.24)	
**Macleod (2008)**	NXY-059	Stroke	Infarct volume	9	725	−0.44 (−0.65, −0.24)	−0.35 (−0.54, −0.17)	−	Mice, rats, rabbits, marmosets
**Pedder (2014)**	Any intervention	Lacunar stroke	Infarct volume	36	563	−0.01 (−0.17, 0.16)	−0.19 (−0.47, 0.09)	−0.00 (−0.17, 0.17)	Rats, rabbits, mice
**Rooke (2011)**	Dopamine	Parkinson's	Neurobehaviour score	601	5800	−0.03 (−0.09, 0.04)	−0.08 (−0.24, 0.08)	−0.08 (−0.14, −0.01)	Mice, rats, monkeys, guinea pigs
**Sena (2007)**	Tirlazad	Stroke	Neurobehaviour score	34	527	−	0.12 (−0.13, 0.36)	−	Rats, rabbits, cats
			Infarct volume	43	651	0.21 (0.03, 0.39)	−0.11 (−0.30, 0.07)	0.15 (−0.06, 0.36)	
									
**Sena (2010)**	Thrombotic occlusion	Stroke	Neurobehaviour score	69	1284	−0.04 (−0.15, 0.07)	−0.06 (−0.21, 0.08)	0.10 (−0.00, 0.22)	Monkeys, rats
			Infarct volume	231	3695	−0.01 (−0.07, 0.05)	−0.03 (−0.11, 0.05)	0.09 (0.02, 0.16)	
**Van der Worp (2007)**	Hypothermia	Stroke	Neurobehaviour score	55	870	−0.16 (−0.30, −0.01)	−	−0.05 (−0.21, 0.10)	Baboons, mice, rabbits, rats
			Infarct volume	222	3256	−0.10 (−0.17, −0.03)	−0.22 (−0.40, −0.03)	−0.06 (−0.13, 0.00)	
**Vesterinen (2010)**	Several interventions	Multiple sclerosis	Neurobehavioural outcomes	3190	64769	−0.01 (−0.03, 0.01)	−	−0.01 (−0.03, 0.01)	Mice, rats, guinea pigs, marmosets, monkeys, ewes
**Vesterinen (2013)**	Rho inhibitors	Stroke	Neurobehaviour score	30	502	0.14 (−0.04, 0.32)	0.09 (−0.20, 0.38)	−0.05 (−0.27, 0.16)	Rats, mice, dogs, gerbils
			Infarct volume	41	654	−0.04 (−0.20, 0.12)	0.00 (−0.29, 0.29)	−0.05 (−0.25, 0.14)	
**Watzlawick (2014)**	RhoA/ROCK-Blockade	Spinal Cord Diseases	Neurobehaviour score	30	655	−0.18 (−0.35, −0.00)	0.03 (−0.14, 0.20)	−0.19 (−0.37, −0.00)	Rats, mice
**Wheble (2007)**	Piracetam	Stroke	Infarct volume	14	197	0.44 (0.16, 0.72)	−	0.34 (0.05, 0.63)	Rats
**Wilmot (2005a)**	NOS inhibitors	Stroke	Neurobehaviour score	16	226	−	−	−0.03 (−0.29, 0.23)	Mice, gerbils, piglets, lambs, cats, rats
			Infarct volume	148	1998	−0.00 (−0.13, 0.12)	−	0.05 (−0.09, 0.20)	
**Wilmot (2005b)**	NOS Donors	Stroke	Infarct volume	40	483	0.09 (−0.10, 0.28)	−	0.01 (−0.34, 0.36)	Rats, rabbits
**Wu (2014)**	Edaravone	Stroke	Neurobehaviour score	30	519	−0.25 (−0.43, −0.08)	−	−0.15 (−0.32, 0.03)	Rats, mice
			Infarct volume	35	503	−0.16 (−0.33, 0.02)	−	−0.01 (−0.22, 0.21)	

* number of animals not reported and not required for analysis.

Twenty systematic reviews investigated treatments for experimental stroke, [17]–[20], [24], [28], [32], [36]–[47] four reviews investigated treatments for spinal cord diseases, [48]–[51] one review each investigated treatments for bone cancer, [52] intracerebral hemorrhage, [39] glioma, [53] multiple sclerosis, [54] Parkinson's disease, [55] and any treatments used in emergency medicine. Animal types included baboons, cats, dogs, ewes, gerbils, guinea pigs, lambs, marmosets, mice, monkeys, pigs, rabbits, rats, and sheep. In our sample 29% of studies reported randomization, 15% reported allocation concealment, and 35% reported blinded outcome assessment.

### 1. Randomization

**Figure 2:**
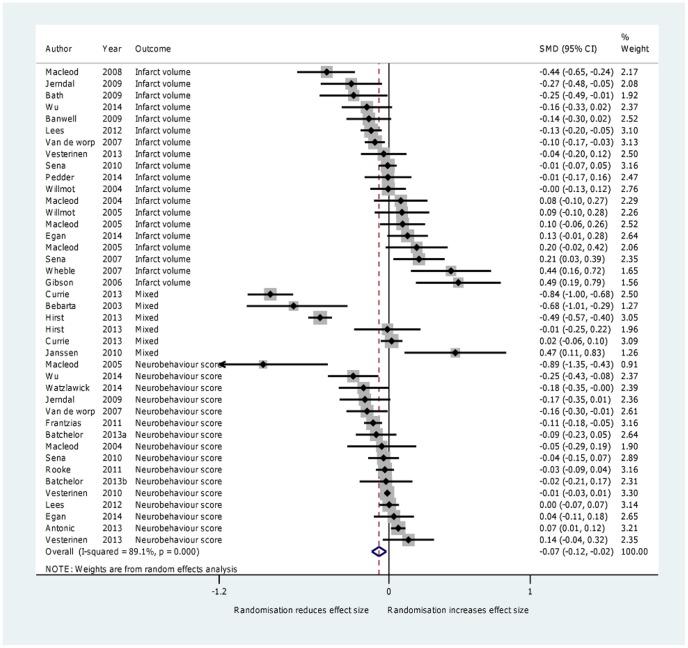
Forest plot showing the effect of random allocation on effect size.

Thirty reviews with 7249 comparisons (121,784 animals) reported the effects of randomization. Randomized trials reduced effect sizes by a moderate and statistically significant amount (SMD  =  −0.07, 95% CI −0.12 to −0.02, I^2^ = 89.1%, *P*  =  0.008) (Figure 2). In a subgroup analysis examining the effect of randomization by disease (stroke versus other), we found that randomization resulted in a lower effect size in areas other than stroke (SMD −0.18, 95% CI −0.30 to −0.06) but not stroke itself (SMD −0.03 95% CI −0.08 to 0.02). However, using meta-regression we found no significant difference between stroke and non-stroke on outcome measures (*P*  = 0.08); additionally, meta-regression could not explain more than 3% of the heterogeneity. A sensitivity analysis excluding the single review [32] in which we had to estimate the number of animals, did not alter the overall result (SMD =  −0.08 95% CI −0.13 to −0.03). In our secondary analysis (where we ignored direction of effect) we found a larger difference between randomized and non-randomized studies (SMD −0.16, 95% CI −0.21 to −0.11, I^2^ = 86.6%, *P*<0.0001) compared with the effect size in which we took direction into consideration.

### 2. Allocation concealment

**Figure 3:**
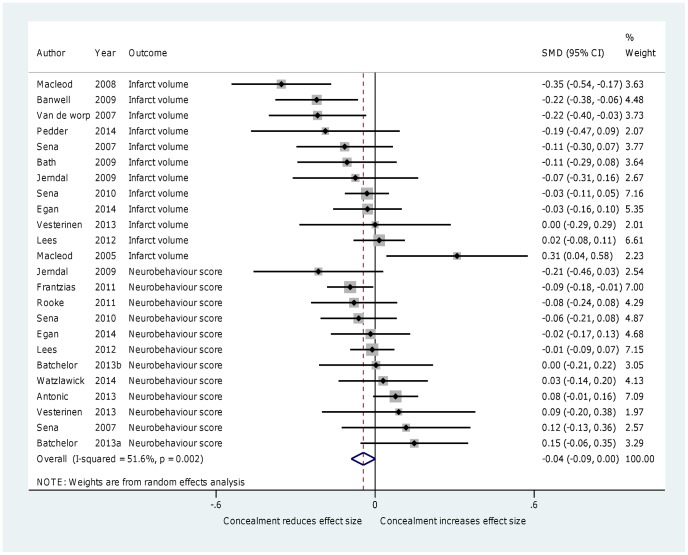
Forest plot showing the effect of allocation concealment on effect size.

Eighteen reviews with 2696 comparisons (39,405 animals) reported the effect of allocation concealment. Studies in which allocation concealment was used resulted in slightly decreased effect sizes, but this was not statistically significant (SMD  =  −0.04, 95% CI −0.09 to 0.00, I^2^ = 51.6%, P = 0.059) (Figure 3). Subgroup analysis examining different diseases (stroke and non-stroke) showed that allocation concealment in studies of stroke resulted in significantly lower effect sizes (SMD =  −0.07, 95% CI −0.12 to −0.02, I^2^ = 48.5%, *P* = 0.009), whereas allocation concealment in other disease areas resulted in higher effect sizes (SMD 0.05, 95% CI −0.01 to 0.11, I^2^ = 0%, *P* = 0.128) but the difference between these groups was not found to be significant using meta-regression (*P* = 0.073). Meta-regression of the combination of disease and outcome measure was did not explain more than 9% of the heterogeneity. In our secondary analysis (where we ignored direction of effect) we found a larger difference between concealed and non-concealed studies (SMD −0.08, 95% CI −0.11 to −0.05, I^2^ = 13.8%, *P*<0.0001) compared with the effect size in which we took direction into consideration.

### 3. Blinding

**Figure 4:**
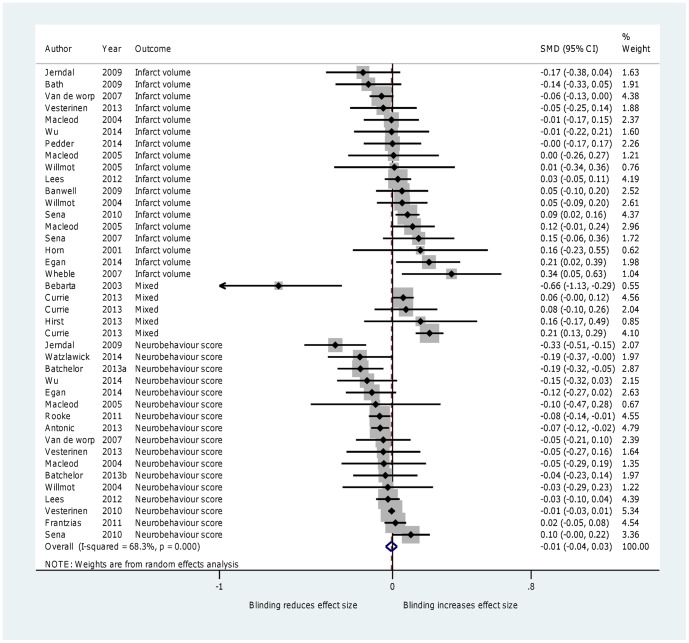
Forest plot showing the effect of blinding of outcome assessment on effect size.

Twenty-eight reviews involving 7140 comparisons (119,597 animals) reported the effects of blinding of outcome assessment. Effect sizes in studies that involved blind outcome assessment were not significantly different from studies that did not (SMD =  −0.01, 95% CI −0.04 to 0.03; I^2^ = 68.3%; *P* = 0.667) (Figure 4). A sensitivity analysis excluding one study in which some estimates were made did not change results. [16] We did not find any differences in effect sizes when we sub-divided studies into stroke and non-stroke groups. In a post-hoc subgroup analysis, we showed that blinding in studies reporting infarct volume did not significantly change effect size (SMD = 0.03, 95% CI −0.02 to 0.08, *P* = 0.187)), whereas blinding in those reporting neurobehavioral outcomes did (SMD =  −0.06, 95% CI −0.10 to −0.02, *P* = 0.003) and this difference was significant when tested using meta-regression (*P* = 0.014). In our secondary analysis (in which effect direction was ignored) we found a larger difference between blinded and non-blinded studies (SMD =  −0.08; 95% CI −0.11, −0.06; I^2^ = 49.5%; *P* < 0.001) compared with the effect size in which we took direction into consideration.

### 4. Risk of bias

Using AMSTAR (Table 2), we found a moderate risk of bias. It was encouraging that all 31 reviews assessed the quality of included studies, all but two reviews used clearly used appropriate methods, and all but two reviews performed comprehensive literature searches. Yet only 9 studies provided a protocol, and only 17 studies searched the grey literature.

**Table 2:** AMSTAR Criteria for included studies*.

	1. Was an 'a priori' design provided?	2. Was there duplicate study selection and data extraction?	3. Was a comprehensive literature search performed?	4. Was the status of publication (i.e. grey literature) used as an inclusion criterion?	5. Was a list of studies (included and excluded) provided?	6. Were the characteristics of the included studies provided?	7. Was the scientific quality of the included studies assessed and documented?	8. Was the scientific quality of the included studies used appropriately in formulating conclusions?	9. Were the methods used to combine the findings of the studies appropriate?	10. Was the likelihood of publication bias assessed?	11. Were conflicts of interest stated?
**Antonic (2013)**	1	1	1	1	1	1	1	1	1	1	1
**Banwell (2009)**	1	2	1	2	1	1	1	1	1	1	2
**Batchelor (2013a)**	1	1	1	2	1	2	1	1	1	1	1
**Batchelor (2013b)**	1	3	1	2	2	2	1	1	1	1	1
**Bath (2009)**	2	2	1	1	1	1	1	1	3	1	1
**Bebarta (2003)**	2	1	2	2	2	2	1	1	1	3	2
**Currie (2013)**	2	1	1	1	1	1	1	1	1	2	1
**Egan (2014)**	2	1	1	2	1	2	1	1	1	1	1
**Frantzias (2011)**	2	1	1	1	2	1	1	1	1	3	1
**Gibson (2006)**	2	1	1	1	1	1	1	1	1	1	2
**Hirst (2013)**	2	1	1	2	2	2	1	1	1	1	1
**Horn (2001)**	2	2	1	1	1	1	1	1	1	1	2
**Janssen (2010)**	2	1	1	2	2	1	1	1	1	2	1
**Jerndal (2010)**	2	1	1	2	1	1	1	1	1	3	1
**Lees (2012)**	2	3	1	2	1	1	1	1	1	1	1
**Macleod (2004)**	2	3	1	1	2	1	1	1	1	1	2
**Macleod (2005a)**	2	3	1	1	2	1	1	1	1	2	2
**Macleod (2005b)**	2	3	1	1	2	1	1	1	1	1	1
**Macleod (2008)**	2	2	1	2	1	1	1	1	3	2	2
**Pedder (2014)**	1	3	1	2	2	1	1	1	1	1	1
**Rooke (2011)**	2	1	1	1	2	2	1	1	1	1	1
**Sena (2007)**	2	1	1	1	2	1	1	1	1	1	2
**Sena (2010)**	2	1	1	1	2	2	1	1	1	1	1
**Van der Worp (2007)**	2	3	1	1	2	2	1	2	1	1	2
**Vesterinen (2010)**	2	1	2	3	2	2	1	1	1	3	1
**Vesterinen (2013)**	1	1	1	2	2	1	1	1	1	1	1
**Watzlawick (2014)**	1	1	1	1	2	2	1	1	1	1	1
**Wheble (2007)**	2	1	1	1	2	1	1	1	1	2	2
**Wilmot (2005a)**	1	1	1	1	2	1	1	1	1	1	2
**Wilmot (2005b)**	2	1	1	1	2	1	1	1	1	1	2
**Wu (2014)**	1	1	1	2	2	2	1	1	1	1	1

*1 = yes, 2 = no, 3 = can't answer, 4 =  not applicable.

## Discussion

In this overview of systematic reviews we found that failure to randomize is likely to result in overestimation of the apparent treatment benefits of interventions across a range of disease areas and outcome measures. We also found a borderline effect of allocation concealment but no overall effect of blinding in our primary analysis. We hypothesize that the reason for an effect of randomization but not allocation concealment or blinding is that subjective judgments are less likely to influence outcomes in trials of (relatively homogeneous) animal models compared with (relatively heterogeneous) humans. While animal heart rates [56], blood flow [57], and behavior can be conditioned by human handling so that placebo controls are sometimes also used in animal studies, [58] there are no ‘patient-reported’ (subjective) outcomes in animal studies. This may make some measures of expectancy effects (for which blinding is useful [5]) smaller in animal studies. Our hypothesis is supported by our post hoc analyses, which showed that blinding reduced effect sizes for (more subjective) neurobehavioral scores, but not for (more objective) infarct volume. It may also be relevant that the comparison of allocation concealment versus non-allocation concealment was reported far less frequently (about half as) as the other comparisons, so the failure to find an effect of allocation concealment could be due to insufficient power. A future individual major study of individual trials is now warranted to investigate the direction, magnitude, and conditions that must hold for randomization, allocation concealment, and blinding to reduce bias in animal studies.

Our results corroborate those of the CAMARADES study, in the sense that we also identified significant bias in animal studies. However, whereas they found a borderline effect of allocation concealment, but no effect for blinding or randomization, we found an effect of randomization, a borderline effect for allocation concealment, and no effect for blinding. The differences between the two reviews could be because our review covered all disease areas, whereas theirs was limited to experimental stroke. In addition, our methods were different; we calculated standardized mean differences rather than (the less widely used and more difficult to replicate) normalized mean differences used by the CAMARADES researchers.

Our study had several potential limitations. First, outcomes, animal models, and disease types were heterogeneous. The high levels of between-study heterogeneity of our overview could not be explained using meta-regression but may result from heterogeneity of the included reviews (and it was beyond the scope of our study to examine the sources of heterogeneity within our included reviews). Secondly, we relied on reports of systematic reviews; these, in turn, relied on reports of individual trials. Some trials may have failed to report randomization, allocation concealment, and blinding when in fact these were used, and vice versa. Evidence from clinical trials suggests that reporting quality is a good surrogate for actual risk of bias. If a similar relationship between reporting quality and study quality in animal studies holds, incomplete reporting may not have affected our results [59]. Based on reporting standards for clinical studies (that require, among other things, descriptions of how randomization, concealment, and blinding were achieved [60]) reporting standards for animal studies have been are emerging. [61] The Animal Research: Reporting In Vivo Experiments (ARRIVE) guidelines, developed in 2010, [62] arguably constitute the leading candidate for becoming a requirement, although development work in this area continues [63]. More recently, it has been suggested that until formal reporting guidelines become required: “at a minimum, authors of grant applications and scientific publications should report on randomization, blinding, sample-size estimation, and the handling of all data”. [61]

Thirdly, it is unclear whether publication bias may have affected our results. It has been estimated that 1 in 6 animal trials remain unpublished, [64] so publication bias may have affected our results. If we assume that unpublished studies were equally likely to be randomized, allocation concealed, and blinded as they were to be non-randomized, not adequately concealed, and unblinded, then publication bias may not have affected the direction of our results. As with human studies, [65] compulsory registration of preclinical studies [66] would reduce publication bias and allow more precise estimates of the empirical dimensions of bias in animal studies.

Fourthly, many of the individual trials included in the systematic reviews applied randomization, allocation concealment, and blinding together, whereas we examined these features independently. Of the 31 included reviews, 19 investigated experimental stroke. If stroke studies tend to be different from other types of studies this might have influenced the results, although we explored this using sub-group analysis and meta-regression. Fifthly, there were a disproportionate number of stroke studies included in out overview of systematic reviews. This was due to the fact that stroke researchers have spearheaded empirical investigations of bias in animal research. Finally, this study was restricted to an investigation of the effects of randomization, allocation concealment, and blinding. Other features, such as lack of power, publication bias, choice of animal models, choice of sex of animals, and choice of outcome may also contribute to the internal and external validity of animal studies. [22], [31], [54], [67] A future individual study systematic review and meta-analysis is now warranted to address these potential limitations.

Our study has implications that extend beyond the conduct of animal studies. Only animal studies that do not suffer from avoidable bias should be accepted as justification for human studies. For this reason, the United States Food and Drug Administration (FDA), [68] the Medical Research Council (MRC) in the United Kingdom, [69] and the World Health Organization (WHO) [70] insist on fair tests, often involving systematic reviews of high quality randomized trials. Our study therefore supports the requirement for adequate conduct and reporting of animal studies, including those being promoted by CAMARADES, and SABRE Research UK. [71]

## Conclusions

Our overview of systematic reviews and meta-analyses revealed that failure to randomize leads to exaggerated effect sizes in animal studies across a wide range of disease areas. In our secondary analysis we found that failure to conceal allocation or employ blind outcome assessment exaggerates effect sizes in animal studies. Biased animal research is less likely to provide trustworthy results, is less likely to provide a rationale for research that will benefit humans, and wastes scarce resources. Requiring compulsory study registration and adherence to emerging evidence-based standards for the conduct and reporting of animal research is likely to reduce the risk of bias in animal studies and improve translatability of animal research.

## Supporting Information

Appendix S1 **Search Strategy.**
(DOCX)

Checklist S1 **PRISMA Checklist.**
(DOC)
