# Supplementary material for: The Need for Randomization in Animal Trials: An Overview of Systematic Reviews
Source: PLoS One. 2014 Jun 6;9(6):e98856. doi: 10.1371/journal.pone.0098856 (PMC4048216; doi:10.1371/journal.pone.0098856)
Supplement: Appendix S1 — Search Strategy. (DOCX) [file pone.0098856.s001.docx]

**Appendix 1. Search Strategy**

**MEDLINE** (OvidSP) [1948 – present]

| \| 1 \| exp animals/ \| 15915049 \| \| --- \| --- \| --- \| \| 2 \| Disease Models, Animal/ \| 186091 \| \| 3 \| animal*.ti. \| 84860 \| \| 4 \| (animal studies or animal experiment?).ab. \| 24928 \| \| 5 \| 1 or 2 or 3 or 4 \| 15928641 \| \| 6 \| limit 5 to "reviews (maximizes specificity)" \| 74588 \| \| 7 \| meta analysis.pt. \| 33031 \| \| 8 \| meta-analysis as topic/ \| 12022 \| \| 9 \| (systematic review or metaanalysis or meta-analysis).ti,ab. \| 57101 \| \| 10 \| (medline or pubmed or embase or biosis or "web of knowledge" or "web of science" or "science citation index").ab. \| 56448 \| \| 11 \| 7 or 8 or 9 or 10 \| 107918 \| \| 12 \| 5 and 11 \| 96120 \| \| 13 \| Publication Bias/ \| 1816 \| \| 14 \| Research Design/ \| 65719 \| \| 15 \| Random Allocation/ \| 73877 \| \| 16 \| double-blind method/ or single-blind method/ \| 129406 \| \| 17 \| therapy.fs. \| 1287748 \| \| 18 \| drug therapy.fs. \| 1521978 \| \| 19 \| Treatment Outcome/ \| 516895 \| \| 20 \| allocat*.ab. \| 54822 \| \| 21 \| conceal*.ab. \| 6109 \| \| 22 \| study design*.ab. \| 97355 \| \| 23 \| quality.ab. \| 404684 \| \| 24 \| blind*.ab. \| 168874 \| \| 25 \| (bias or unbias* or un-bias*).ab. \| 68229 \| \| 26 \| efficacy.ab. \| 371164 \| \| 27 \| intervention?.ab. \| 396739 \| \| 28 \| (full adj5 abstract?).ab. \| 773 \| \| 29 \| (heterogeneity or heterogeneous).ab. \| 148315 \| \| 30 \| Data Interpretation, Statistical/ \| 41830 \| \| 31 \| "Reproducibility of Results"/ \| 228477 \| \| 32 \| 13 or 14 or 15 or 16 or 17 or 18 or 19 or 20 or 21 or 22 or 23 or 24 or 25 or 26 or 27 or 28 or 29 or 30 or 31 \| 4267171 \| \| 33 \| 6 and 32 \| 53993 \| \| 34 \| 12 and 32 \| 67617 \| \| 35 \| human/ \| 12214445 \| \| 36 \| 33 not 35 \| 311 \| \|  \|  \|  \| |
| --- | --- | --- | --- | --- | --- | --- | --- | --- | --- | --- | --- | --- | --- | --- | --- | --- | --- | --- | --- | --- | --- | --- | --- | --- | --- | --- | --- | --- | --- | --- | --- | --- | --- | --- | --- | --- | --- | --- | --- | --- | --- | --- | --- | --- | --- | --- | --- | --- | --- | --- | --- | --- | --- | --- | --- | --- | --- | --- | --- | --- | --- | --- | --- | --- | --- | --- | --- | --- | --- | --- | --- | --- | --- | --- | --- | --- | --- | --- | --- | --- | --- | --- | --- | --- | --- | --- | --- | --- | --- | --- | --- | --- | --- | --- | --- | --- | --- | --- | --- | --- | --- | --- | --- | --- | --- | --- | --- | --- | --- | --- | --- |

**Embase** (OvidSP) [1974 – present]

Top of Form

| \| 1 \| animal model/ \| 633958 \| \| --- \| --- \| --- \| \| 2 \| animal experiment/ \| 1607367 \| \| 3 \| (nonhuman/ or animal*.ti.) and disease model/ \| 22479 \| \| 4 \| animal*.ti. \| 96334 \| \| 5 \| (animal studies or animal experiment?).ti,ab. \| 37034 \| \| 6 \| 1 or 2 or 3 or 4 or 5 \| 1826626 \| \| 7 \| limit 6 to "reviews (maximizes specificity)" \| 767 \| \| 8 \| allocat*.ab. \| 66424 \| \| 9 \| conceal*.ab. \| 7670 \| \| 10 \| study design*.ab. \| 112606 \| \| 11 \| quality.ab. \| 531150 \| \| 12 \| blind*.ab. \| 214727 \| \| 13 \| (bias or unbias* or un-bias*).ab. \| 78351 \| \| 14 \| efficacy.ab. \| 486204 \| \| 15 \| intervention?.ab. \| 501009 \| \| 16 \| (full adj5 abstract?).ab. \| 886 \| \| 17 \| (heterogeneity or heterogeneous).ab. \| 174730 \| \| 18 \| treatment outcome/ \| 582189 \| \| 19 \| outcome assessment/ \| 158884 \| \| 20 \| randomization/ \| 57860 \| \| 21 \| 8 or 9 or 10 or 11 or 12 or 13 or 14 or 15 or 16 or 17 or 18 or 19 or 20 \| 2461736 \| \| 22 \| 7 and 21 \| 372 \| |  |
| --- | --- | --- | --- | --- | --- | --- | --- | --- | --- | --- | --- | --- | --- | --- | --- | --- | --- | --- | --- | --- | --- | --- | --- | --- | --- | --- | --- | --- | --- | --- | --- | --- | --- | --- | --- | --- | --- | --- | --- | --- | --- | --- | --- | --- | --- | --- | --- | --- | --- | --- | --- | --- | --- | --- | --- | --- | --- | --- | --- | --- | --- | --- | --- | --- | --- | --- | --- |
